# Aquatic Exercise Positively Affects Physiological Frailty among Postmenopausal Women: A Randomized Controlled Clinical Trial

**DOI:** 10.3390/healthcare9040409

**Published:** 2021-04-02

**Authors:** Ji-Hyeon Kim, Min-Seong Ha, Soo-Min Ha, Do-Yeon Kim

**Affiliations:** 1 Department of Liberal Arts, Mokpo National Maritime University, Jeollanam-do 58628, Korea; frogmankjh@mmu.ac.kr; 2 Department of Sports Culture, College of the Arts, Dongguk University-Seoul, Seoul 04620, Korea; haminseong@dgu.ac.kr; 3 Laboratory of Exercise Physiology, Department of Physical Education, Pusan National University, Busan 46241, Korea; fantasista@pusan.ac.kr

**Keywords:** aging-related hormones, aquatic exercise, cardiovascular disease, elderly women, insulin resistance, physiological frailty

## Abstract

Frailty is a risk factor associated with aging. Physical exercise is an important lifestyle factor that can help to avoid risks associated with aging. Therefore, we aimed to determine the effects of aquatic exercise for 12 weeks on body composition, cardiovascular disease risk factors, insulin resistance, and aging-related sex hormones in elderly South Korean women. Twenty-two women aged 70–82 years were randomly assigned to groups that participated or did not participate (controls; *n* = 10 in aquatic exercise for 60 min, three times per week for 12 weeks (*n* = 12). Exercise intensity defined as the rating of perceived exertion (RPE), was increased from 12–13 to 13–14, and to 14–15 during weeks 1–4, 5–8, and 9–12, respectively. Body composition (skeletal muscle mass, ratio (%) body fat, and waist circumference), cardiovascular disease risk factors (total, high-density lipoprotein, and low-density lipoprotein cholesterol), insulin resistance (glucose, insulin, and homeostatic model assessment of insulin resistance [HOMA-IR]), and aging-related sex hormone changes (dehydroepiandrosterone-sulfate [DHEA-S]) and sex hormone-binding globulin [SHBG]) were assessed. Aquatic exercise safely improved body composition, reduced insulin resistance, and positively affected the sex hormones DHEA-S and SHBG as well as blood lipid profiles. Our findings suggested that the aquatic exercise program positively altered blood lipids, regulated glucose levels, and sex hormone levels. Therefore, regular, and continuous aquatic exercise is recommended to prevent frailty, decrease cardiovascular risk, and provide older women with an optimal quality of life as they age.

## 1. Introduction

In the face of rapidly increasing population aging around the world, frailty is a representative expression of major implications for clinical practice and public health [1,2]. The condition of frailty is characterized by reducing multiple physiological systems such as being vulnerable to poor resolution of homeostasis by an increased vulnerability to stressors [1,2,3]. Furthermore, frailty among the elderly population results in the inability to participate in normal levels of physical activity. Decreased physical activity significantly impacts the incidence of chronic diseases [4]. Women are more likely than men to develop frailty, which progresses with aging and increases mortality because of reduced fat-free mass (FFM) and muscle strength [3]. Prevalent changes in body composition during the early stages of frailty include decreased muscle mass, skeletal muscle weakness, and increased fat mass [5]. Decreased FFM leads to increased metabolic dysregulation at rest [6], diminished muscle function, increased insulin resistance, and increased incidence of obesity and cardiovascular disease (CVD) [7]. Ultimately, frailty is likely to facilitate aging, as frail individuals undergo rapid health deterioration under even minor amounts of stress [1].

Hypertension, which is associated with frailty, is a significant vascular disease that can lead to life-threatening conditions such as coronary artery disease, stroke, and peripheral vascular disease [8]. Postmenopausal decreases in ovarian hormones among older women result in increased low-density lipoprotein cholesterol (LDL-C) and triglyceride (TG), decreased high-density lipoprotein cholesterol (HDL-C), and increased insulin levels because of increased insulin resistance. Postmenopausal loss of sex hormones eventually increases risk of cardiovascular and metabolic diseases [9].

Reductions in levels of hormones including estradiol, testosterone, dehydroepiandrosterone sulfate (DHEA-S), and sex hormone-binding globulin (SHBG) lead to diminished muscle function, loss of spinal motor neurons, declining physical functions, and motor abnormalities in aging women [10,11]. Estradiol, the primary female hormone and the most abundant of the circulating estrogens, is associated with CVD prevention in women through increased angiogenesis and vasodilation, and decreased reactive oxygen species, oxidative stress, and fibrosis [12]. Testosterone, an essential hormone in women that directly or indirectly interacts with estradiol throughout the body [13], plays a pivotal role in cardiovascular function in both men and women [14]. Reduced total testosterone levels are associated with a higher incidence of CVD in older women [15].

Levels of DHEA-S start to decline during early adulthood, decreasing by 80–90% by the age of 70–80 years [11]. Low serum DHEA-S levels increase the incidence and mortality of vascular diseases among elderly persons [16] and are associated with the development of atherosclerosis [17]. The protein SHBG transports sex steroid hormones with a higher affinity for testosterone than estradiol [18]. As serum SHBG levels are inversely proportional to body weight, increased body weight changes sex hormone levels due to decreased SHBG levels. Lower SHBG levels are associated with insulin resistance, thus rendering this protein an important metabolic marker of increased risk for diabetes [19,20,21]. The relationship between SHBG levels and insulin resistance is unrelated to estrogen and testosterone levels [22], and SHBG is closely associated with CVD [23]. These findings suggest that aging-related sex hormone changes lack complexity and have a close, independent relationship with cardiovascular risk factors. Therefore, increasing the secretion of age-related hormones should protect against CVD and help to delay signs of aging. Hence, this study investigated changes in aging-related sex hormone changes caused by exercise.

Aerobic and resistance exercise can prevent loss of muscle mass and declining muscular function in elderly individuals [24]. It is also one of the best ways to prevent and treat hypertension, a leading risk factor for CVD [8]. The American College of Sports Medicine (ACSM) and the American Heart Association (AHA) recommend aerobic and resistance exercises to improve cardiorespiratory and muscular function [25]. Aquatic exercise is a simple way of exercising for older women, as water resistance enhances cardiorespiratory and muscular function. Aquatic environment cools the body and lowers the heart rate by 10–13 bits compared to usual, which affects the gravity and reduces the compression force of the body, thereby reducing the demand for the CV system. Furthermore, lower gravitational force, which results in minimal shock to muscles and joints during exercise. This type of aerobic exercise can be practiced for rehabilitation and to prevent joint injury, and thus is recommended for elderly persons [26,27].

In the previous study, we applied aquatic exercise to healthy elderly people and obtained positive results by studying their physiological changes in various angles [4,26,28,29]. Based on these results, we intended to verify the effectiveness of aquatic exercise on physiological frailty, focusing on changes in blood lipids, insulin resistance, and aging-related changes. Thus, this study aimed to determine the effects of 12 weeks of aquatic exercise on body composition, cardiovascular risk factors, insulin resistance, and aging-related hormones that are closely associated with frailty, in healthy South Korean women aged 70–82 years who did not exercise regularly.

## 2. Materials and Methods

### 2.1. Study Design and Participants

**Figure 1:**
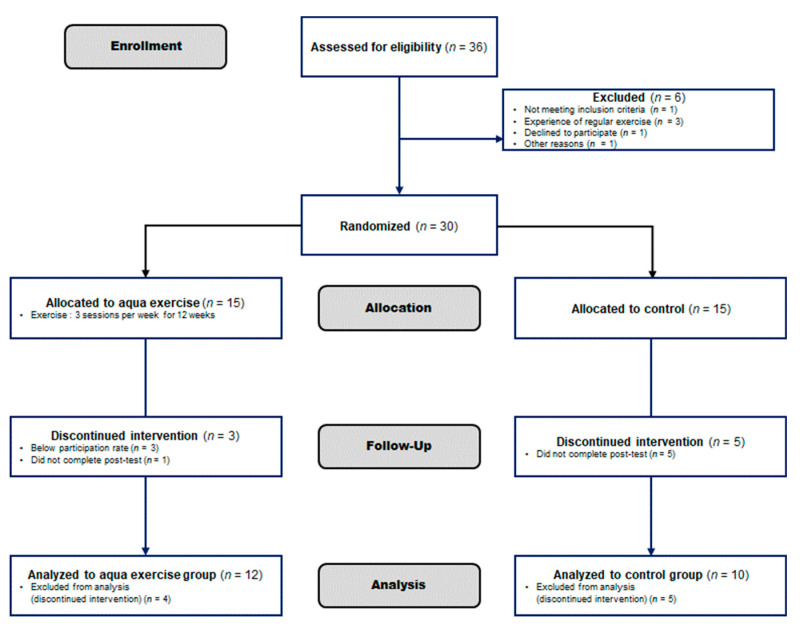
Study flow chart based on Consort 2010 Flow Chart Diagram. The first 36 elderly women were enrolled for our experiment, but 6 were excluded because they were not suitable for the study. Thirty participants were randomly assigned to the Aquatic exercise group and the Control group, and the final subjects were Aquatic exercise (*n* = 12) vs. the Control group (*n* = 10).

This randomized controlled study was conducted in Busan, South Korea, for 12 weeks in women aged 70–82 years. The exercise group participated in aquatic exercises three times a week, and the control group maintained their usual activities. The outcomes were assessed at baseline and at the end of the exercise program in the following order: body composition (weight; body mass index, BMI; skeletal muscle mass, SMM; body fat percentage, %BF; waist circumference, WC), CVD risk factors (systolic blood pressure, SBP; diastolic blood pressure, DBP; total cholesterol, TC; TG, HDL-C, LDL-C), insulin resistance (glucose, insulin, homeostatic model assessment for insulin resistance, HOMA-IR), and aging-related sex hormones (estradiol, testosterone, DHEA-S, and SHBG). This study was approved by national bioethics committee (PNU IRB) and was performed in accordance with the Helsinki Declaration and its later amendments. It was conducted as a derivative study of PNU IRB/2017_68_HR and the 2010 Consolidated Standards of Reporting Trials statement [30]. The optimal number of participants was derived using G-power version 3.1 for Windows (Kiel University, Kiel, Germany) [31,32]. The initial sample size was calculated to be 34 based on the repeated measures (ANOVA) F-statistic with significance at *p* < 0.05, a default effect size of 0.25 and power of 80%. We selected 36 participants while considering that some would drop out. We finally randomly assigned 30 women to exercise or control groups (*n* = 15 each). By the end of the study, 12 and 10 participants remained in the exercise and control groups, respectively, and the power of the test was 60%. Figure 1 shows the flow chart of the study. The inclusion criteria comprised women who had not exercised regularly for the last 6 months, and could walk, and participate in physical activity without the need for equipment. The exclusion criteria were: under medication, or supplements during the study, changed dietary and exercise habits during the study, consumed an unbalanced diet, or excessive alcohol, and refrained from participation for long periods.

### 2.2. Aquatic Exercise Program

The aquatic exercise program was developed by combining and improving the programs described by Ha et al. [4] and Kim et al. [29]. The average water temperature was 28–30 °C. Considering that the participants were aged 70–82 years, the program was preceded by a 1–2-week adjustment period, followed by three sessions per week for 12 weeks. Each session included a warm-up, 10 min; main exercise, 40 min, and cool-down, 10 min. The exercise intensity was set at rating of perceived exertion (RPE) of 9–10, 11–12 and 13–14 for weeks 1–4, 4–8, and 9–12, respectively [33]. The participants also wore a heart rate monitor (Polar RS400sd, USA) to ensure that exercise intensity was maintained at 30–60% of the heart rate reserve. Supplemental Table S1 shows details of the aquatic exercise program.

### 2.3. Body Composition

Height, weight, BMI, SMM, and %BF were measured using the bioelectrical impedance analysis tool, X-SCAN PLUS II (Jawon Medical, Seoul, Korea), with the participants wearing simple clothing, as recommended by the ACSM [34]. Waist circumference was measured at the midpoint between the lowest point of the rib cage and the ilium in upright participants [35].

### 2.4. Blood Pressure

We measured the means of two measurements each of SBP and DBP using a mercury sphygmomanometer (Baumanometer; W.A. Baum Co., Inc., Copiage, NY, USA) after a 20-min rest.

### 2.5. Biochemistry Analysis

Blood (10 mL) was collected from the forearm veins of participants after fasting for at least 10 h between 8 and 9 a.m. Samples were placed in serum separator tubes containing ethylenediaminetetraacetic acid, separated by centrifugation (Combi-514R; Hanil, Korea) at 3000 rpm for 15 min, then stored at –70 °C. Levels of TC, TG, HDL-C, and LDL-C were analyzed using an ADVIA^®^1650 chemistry system (Siemens Healthcare, Tarrytown, NY, USA), and reagents from Siemens (Siemens Healthcare, Tarrytown, NY, USA). Fasting blood glucose levels were measured using a Toshiba TBA 200FR NEO analyzer (Diamond Diagnostics Inc., Holliston, MA, USA). Insulin levels were analyzed using insulin electrochemiluminescence (ECL) immunoassays (Roche Diagnostics GmbH., Mannheim, Germany) and a Roche Modular E170 immunoassay module (Roche Diagnostics GmbH., Mannheim, Germany) We calculated the HOMA-IR as (fasting insulin [μU/mL] × fasting blood glucose [mg/dL])/405.

Estradiol levels were analyzed using ECL estradiol III kits (Roche Diagnostics GmbH., Mannheim, Germany) and Roche Modular E170 immunoassay module (Roche Diagnostics GmbH., Mannheim, Germany). Testosterone levels were analyzed using Coat-A-Count and DHEA-S kits (Diagnostic Products Corporation, Los Angeles, CA, USA). Levels of SHBG were analyzed using IRMA-Count SHBG kits (Diagnostic Products Corporation) and a Cobra 5010 II Gamma Counter (Packard Instrument Co., Inc., Meriden, CT, USA).

### 2.6. Data Analysis

Data were analyzed using SPSS Statistics version 23.0 (IBM Corp., Armonk, NY, USA). Means and standard deviations were calculated, and group and time-treatment interaction effects were assessed by two-way repeated-measure ANOVA, followed by Bonferroni’s multiple comparison tests for post-hoc analysis. One-way ANOVA with Dunnett’s multiple comparison tests was used to analyze the delta (Δ) change. It was recommended that an ANCOVA be utilized with the pre-test values as covariates. The data were analyzed with ANCOVA with the contrast of adjusted mean values, inserting the baseline result of each variable as a covariate. Values with α = 0.05 were considered statistically significant. Effect sizes (Cohen’s *d*) between pre- and post-test data were expressed as mean changes [32].

## 3. Results

### 3.1. Participant Characteristics

Table 1 shows the characteristics of the participants. Risk factors for CVD, aging-related hormones, and body composition were measured before and after 12 weeks of aquatic exercise.

### 3.2. Body Composition

**Figure 2:**
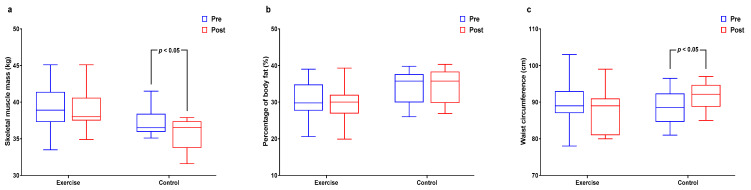
Effect of 12 weeks of aquatic exercise on body composition in elderly Korean women. (**a**) SMM of control decreased significantly (*p* < 0.05); (**b**) % BF showed significant interaction effects (*p* < 0.05); (**c**) WC of control increased significantly (*p* < 0.05). SMM: skeletal muscle mass, % BF: percentage of body fat, WC: waist circumference.

Figure 2 and Supplemental Table S2 shows interactions and differences within and between groups according to body composition. Whereas body weight and BMI did not significantly differ, SMM had significant interaction and main group effects (*p* < 0.01 for both) and significantly decreased in the control group (*p* < 0.05). The %BF had significant interaction effects (*p* < 0.05) and WC had significant interaction effects (*p* < 0.05) and significantly increased in the control group (*p* < 0.05).

#### ANCOVA Analysis of SMM

Table 2 show the pre-value of SMM was treated as a covariate, it was found that there was a difference in the post-value of SMM according to the group (F = 9.542, *p* < 0.01). In addition, the pre-value of SMM set as a covariate was found to have a significant relationship with the post-value of SMM, and the exercise group was more involved in the SMM-post value than the control group. In addition, the total predicted variance affecting the posterior value of SMM was 0.669 (corrected R^2^ = 0.632).

### 3.3. Cardiovascular Risk Factors

**Figure 3:**
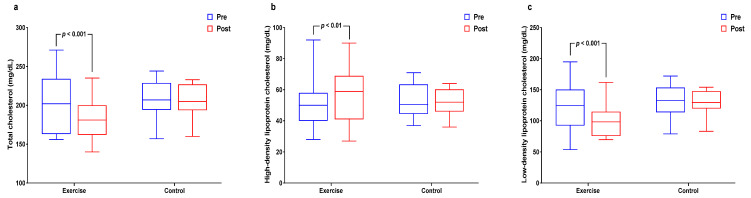
Effect of 12 weeks of aquatic exercise on cardiovascular risk factors in elderly Korean women. (**a**) TC of the exercise group significantly decreased (*p* < 0.001); (**b**) HDL-C of exercise group increased significantly (*p* < 0.01); (**c**) LDL-C of the exercise group was significantly decreased (*p* < 0.001). TC: total cholesterol, HDL-C: high-density lipoprotein cholesterol, LDL-C: low-density lipoprotein cholesterol.

Figure 3 and Supplemental Table S3 show interactions and differences within and between groups according to cardiovascular risk factors. Significant interaction and main time effects (*p* < 0.01 for both) were found for TC levels along with a significant decreased in the exercise group (*p* < 0.01). Interaction effects were significant (*p* < 0.05) for HDL-C along with a significant increase in the exercise group (*p* < 0.05). Significant interaction (*p* < 0.05) and time (*p* < 0.01) effects were found for LDL-C levels, along with a significant decrease in the exercise group (*p* < 0.01).

### 3.4. Insulin Resistance

**Figure 4:**
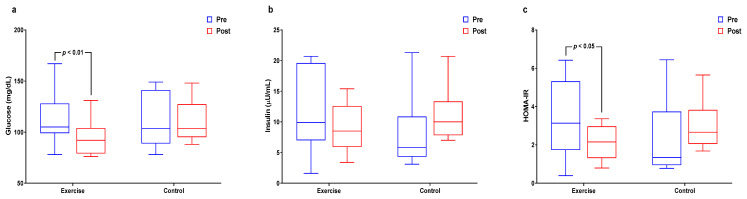
Effect of 12 weeks of aquatic exercise on insulin resistance in elderly Korean women. (**a**) The glucose of the exercise group decreased significantly (*p* < 0.01); (**b**) Insulin showed significant interaction effects (*p* < 0.05); (**c**) HOMA-IR of the exercise group was significantly decreased (*p* < 0.05). HOMA-IR: homeostatic model assessment for insulin resistance.

Figure 4 and Supplemental Table S4 show the interactions and differences within and between groups for insulin resistance. Glucose levels had significant interaction effects and significantly decreased in the exercise group (*p* < 0.05 for both). The insulin level had significant interaction effects (*p* < 0.05) and HOMA-IR had significant interaction effects and significantly decreased in the exercise group (*p* < 0.05 for both).

### 3.5. Aging-Related Sex Hormones

**Figure 5:**
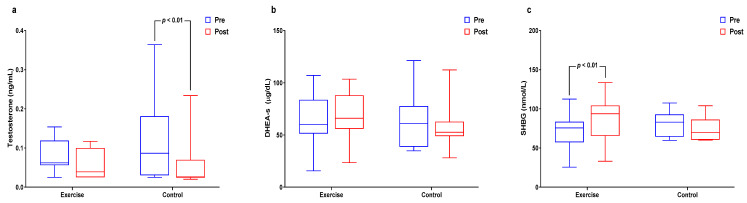
Effect of 12 weeks of aquatic exercise on aging-related sex hormones in elderly Korean women; (**a**) Testosterone of the control group was significantly decreased. (*p* < 0.01); (**b**) DHEA-S showed significant interaction effects (*p* < 0.05); (**c**) SHBG was significantly increased in the exercise group (*p* < 0.01). SHBG: sex hormone-binding globulin.

Figure 5 and Supplemental Table S5 shows within- and between-group interactions and differences in aging-related sex hormone levels. The main time effect was significant for testosterone levels (*p* < 0.001), which significantly decreased in the control group (*p* < 0.01). The interaction effect was significant (*p* < 0.05) for DHEA-S levels. Interaction effects were significant (*p* < 0.01) for SHBG levels, which significantly increased in the exercise group (*p* < 0.01).

### 3.6. Difference (Δ) in All Valuables after Aquatic Exercise Intervention

**Figure 6:**
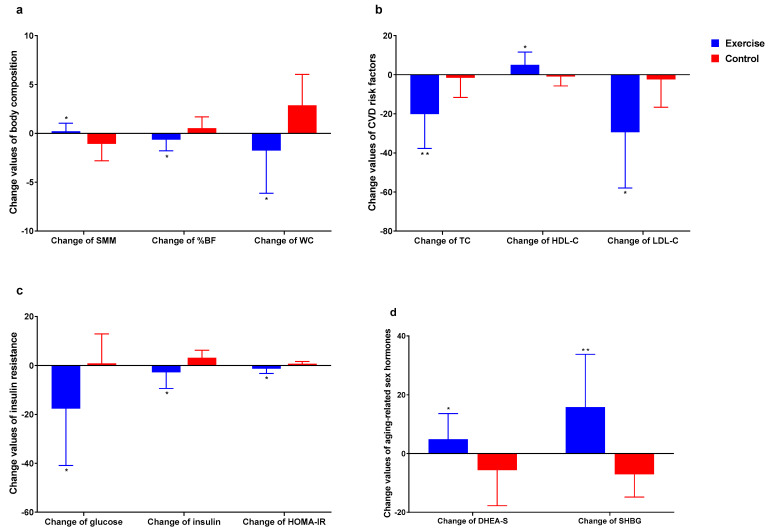
Difference in the change value of each of the variables. (**a**) Body composition; (**b**) Cardiovascular disease risk factors; (**c**) Insulin resistance. (**d**): Aging-related sex hormones. * *p* < 0.05, ** *p* < 0.01.

Figure 6 shows difference in the change value of each of the variables. Briefly, Δ-SMM was found to be higher in exercise group (*p* < 0.05) compared to control group. Δ-%BF and Δ-WC were found to be lower in exercise group (*p* < 0.05) compared to control group. Δ-TC and Δ-LDL-C level were found to be lower in exercise group (*p* < 0.01, *p* < 0.05) compared to control group. HDL-C level was found to be higher in exercise group (*p* < 0.05) compared to control group. Δ-Glucose, Δ-Insulin and Δ-HOMA-IR level were found to be lower in exercise group (*p* < 0.05) compared to control group. Δ-DHEA-S and Δ-SHBG level were found to be higher in exercise group (*p* < 0.05, *p* < 0.01) compared to control group.

## 4. Discussion

Muscle mass becomes more difficult to maintain with aging [36], and the decrease in FFM and increase in body fat mass need particular attention [37,38]. Although body weight and BMI did not significantly change in either group, the exercise group maintained skeletal muscle mass and reduced %BF.

Aquatic exercise is recommended as an alternative to ground exercise for older women who are unable to tolerate excessive weight-bearing exercises [39]. Aquatic exercise can help to maintain proper body composition because the effects on exercise capacity increase with increasing water resistance [40]. The present findings suggested that aquatic exercise positively helps to maintain skeletal muscle mass and reduces %BF. Aquatic exercise enhances cardiorespiratory function [41], thus increasing aerobic capacity and optimizing skeletal muscle performance in elderly women.

Epidemiologic studies have shown that LDL-C, which is a potent risk factor for CVD, is a major cause of atherosclerosis [42]. While HDL-C weakens the relationship, elevated TG and LPL levels increase the risk of CVD [34]. Frailty is significantly associated with TC, LDL-C, HDL-C, and fasting blood glucose levels [43]. Serum TC and LDL-C levels significantly decreased, whereas HDL-C levels significantly increased in elderly South Korean women after 12 weeks of aquatic exercise. These findings indicated that regular aquatic exercise would help to reduce risks for CVD, such as coronary artery disease or arteriosclerosis, and prevent frailty by delaying aging-related changes in lipid levels.

Most participants in this study had risk factors for prehypertension or hypertension stage 1. The estradiol levels were deficient in the aquatic exercise and control groups (5.46 ± 0.72 and 5.71 ± 0.68 pg/mL, respectively). The prevention and management of hypertension are particularly important for older women because of blood pressure rapidly elevates due to a postmenopausal estrogen deficiency. Estrogen prevents atherosclerosis, and levels are higher in women with normal vasculature than in those with atherosclerosis [44].

Aquatic exercise can be safely prescribed for overweight and obese hypertensive women because it does not cause a rapid post-exercise decrease or increase in blood pressure [45]. Water and ground exercise are equally effective in terms of lowering blood pressure in postmenopausal hypertensive women [46]. Estradiol levels did not significantly change after 12 weeks of aquatic exercise, but tended to be more pronounced in the control, than the exercise group. The decrease in blood pressure also tended to be more significant in the exercise group than the control group. These results might reflect a slow reduction in estradiol levels and a decrease in hypertension caused by aquatic exercise.

Exercise promotes glucose uptake and mitochondrial function and increases insulin sensitivity [47,48]. Older women with low levels of physical activity who participate in an aquatic exercise program have decreased levels of free fatty acid, which inhibits insulin action, thus lowering insulin resistance [49].

Insulin resistance decreased in our aquatic exercise group due to post-exercise reductions in fasting blood glucose and insulin levels. Physical activity increases glucose transporter type 4 in muscle and induces protein kinase B (PKB; Akt) phosphorylation by increasing insulin that decreases glucose levels [50]. Physical activity also increases glucose uptake by stimulating muscle membranes and cells [48] and improves insulin sensitivity by enhancing insulin resistance through increased muscle mass and reduced fat mass [51]. Thus, regular aquatic exercise apparently enhances insulin binding in muscle cells and regulates blood glucose levels with low insulin levels.

Women have overall low testosterone levels, and increased testosterone utilization increases cardiovascular risk [15]. Since DHEA is converted to DHEA-S in the liver and then to estrogen, which is more active in adrenal and peripheral tissues [52], it might affect various body systems and help prevent the deleterious effects of aging [53]. High levels of DHEA-S are associated with increased muscle strength, bone density, and anti-inflammatory activity as well as better immunoregulation [54]. Conversely, low DHEA-S decreases levels of anabolic steroids, such as androstenedione, testosterone, and estrogen [55]. Furthermore, DHEA-S is associated with cardiovascular mortality at a certain age threshold [56].

Obesity and metabolic endpoints are more closely associated with SHBG than either estrogen or testosterone [57] and SHBG positively correlate with HDL-C levels [58]. Levels of DHEA-S were significantly higher in our exercise, than in the control group. The SHBG and HDL-C levels significantly increased, and insulin resistance significantly decreased in our exercise, compared with the control group. Moreover, body weight did not significantly change in either group during the study. Levels of SHBG decrease in the presence of weight gain, and of insulin resistance and hyperinsulinism, regardless of weight. We also found higher glucose, insulin, and HOMA-IR levels and significantly lower SHBG levels in the control group, than in the aquatic exercise group. Therefore, reductions in body weight, insulin levels, or HOMA-IR levels could explain the increase in SHBG found in elderly South Korean women. Because low SHBG is associated with high insulin resistance and high insulin values regardless of weight, low SHBG levels might serve as an indicator of hyperinsulinism and insulin resistance. That is, low SHBG can be a predictor of type II diabetes [59]. Therefore, increased DHEA-S and SHBG levels caused by regular aquatic exercise should help to prevent CVD and reduce risk for metabolic diseases in women aged 70–82 years.

Several limitations of the present study should be acknowledged. First, the small number of participants does not allow for generalization. However, this study may be an important conceptual basis to explore the effects of aquatic exercise on elderly women in future, larger experiments. Second, our participants were healthy elderly women, and thus our results might not be generalizable to other populations. Third, although we do not recommend changing activities of daily living, the dietary status of the elderly women in the present study was not assessed, which might have influenced the results. Further studies are warranted to address these issues.

## 5. Conclusions

Aquatic exercise improves body composition and positively affected blood lipid profiles that are cardiovascular risk factors among elderly women. Aquatic exercise is also considered effective in reducing insulin resistance and metabolic and cardiovascular risks by increasing DHEA-S and SHBG levels. Therefore, regular, ongoing aquatic exercise is recommended for older women to improve their quality of life during aging, and prevent frailty.

## Figures and Tables

**Table 1:** Physical characteristics of participants in each group.

	Variables	Age (y)	Height (cm)	Weight (kg)	BMI (kg/m^2^)	SMM (kg)	% BF (%)
Group							
Exercise(*n* = 12)		74.36 ± 3.78	154.27 ± 3.93	56.54 ± 7.38	23.73 ± 2.19	21.65 ± 1.64	30.41 ± 5.53
Control(*n* = 10)		75.90 ± 4.23	153.00 ± 4.08	58.45 ± 2.44	24.99 ± 1.11	20.18 ± 1.23	34.31 ± 4.45
*t*		−0.880	0.728	0.812	−1.816	2.293 *	1.768

Values are mean ± standard deviation. * *p* < 0.05. BMI: body mass index, SMM: skeletal muscle mass, % BF: percentage of body fat.

**Table 2:** ANCOVA analysis of SMM.

Variable			Mean Square	F	R^2^
SMM-pre (kg)			14.861	9.898 **	0.669
SMM-post (kg)	Exercise (*n* = 12)	21.85 ± 1.83	14.327	9.542 **	
	Control (*n* = 10)	19.10 ± 0.98			

Values are mean ± standard deviation. ** *p* < 0.01.

## Data Availability

Not applicable.

## Supplementary Materials

The following are available online at https://www.mdpi.com/article/10.3390/healthcare9040409/s1, Table S1: Aquatic exercise program, Table S2: Changes in body composition after a 12-week aquatic exercise program, Table S3: Changes in cardiovascular disease risk factors after a 12-week aquatic exercise program, Table S4: Changes in insulin resistance after a 12-week aquatic exercise program, Table S5: Changes in aging-related sex hormones after a 12-week aquatic exercise program.
