# Supplementary material for: Aquatic Exercise Positively Affects Physiological Frailty among Postmenopausal Women: A Randomized Controlled Clinical Trial
Source: Healthcare (Basel). 2021 Apr 2;9(4):409. doi: 10.3390/healthcare9040409 (PMC8065774; doi:10.3390/healthcare9040409)
Supplement: Supplementary file 1 [file healthcare-09-00409-s001.pdf]

Supplemental Table 1. Aquatic exercise program

Week	Order	Description of aquatic exercise	Intensity
1-12	Warm-up<br>(10 min)	Stretching<br>Slow, wide walking<br>Bouncing	
1-4	Main exercise<br>(40 min)	Aquatic step box<br>Step-up<br>Side stepping<br>Hopping	40%-50% HRR (RPE 12-13)
5-8		Hydro-tone<br>Resistive walking<br>Scissors	50%-60% HRR (RPE 13-14)
9-12		Powerful, fast jogging<br>Kicking	60%-70% HRR (RPE 14-15)
1-12	Cool-down<br>(10 min)	Stretching<br>Slow, wide walking<br>Bouncing	

HRR: heart rate reserve, RPE: ratings of perceived exertion.

Supplemental Table 2. Changes in body composition after a 12-week aquatic exercise program

Variable	Group	Pre	Post	△	*F*		**Effect size Cohen's *d***
Weight (kg)	Exercise (n=12)	56.54 ±7.38	56.11 ±7.68	-0.43 ±1.34	Group	0.800	-0.06
					Time	0.183	
	Control (n=10)	58.45 ±2.44	58.67 ±2.77	0.22 ±0.78	Interaction	1.781	0.09
BMI (kg/m^2)	Exercise (n=12)	23.73 ±2.19	23.55 ±2.35	-0.18 ±0.58	Group	3.276	-0.08
					Time	0.166	
	Control (n=10)	24.99 ±1.11	25.07 ±0.94	0.08 ±0.32	Interaction	1.671	0.07
SMM (kg)	Exercise (n=12)	21.65 ±1.64	21.85 ±1.83	0.21 ±0.82	Group	13.456^{**}	0.12
					Time	2.261	
	Control (n=10)	20.18 ±1.23	$19.10^{\#}$ ±0.98	-1.08 ±1.72	Interaction	4.954^{*}	-0.06
% BF (%)	Exercise (n=12)	30.41 ±5.53	29.77 ±5.61	-0.64 ±1.14	Group	4.091	-0.12
					Time	0.053	
	Control (n=10)	34.31 ±4.45	34.83 ±4.59	0.52 ±1.17	Interaction	5.276^{*}	0.12
WC (cm)	Exercise (n=12)	90.23 ±6.31	88.45 ±6.23	-1.77 ±4.37	Group	0.143	-0.28
					Time	0.419	
	Control (n=10)	88.75 ±4.72	$91.61^{\#}$ 3.87	2.86 ±3.17	Interaction	7.600^{*}	0.61

Values are mean±standard deviation. BMI: body mass index, SMM: skeletal muscle mass, % BF: percentage of body fat, WC: waist circumference. $^{*}p < 0.05$, $^{**}p < 0.01$, $^{\#}p < 0.05$. Effect size range: |0.20|≦small<|0.50|<medium<|0.80|≦large.

Supplemental Table 3. Changes in cardiovascular disease risk factors after a 12-week aquatic exercise program

Variable	Group	Pre	Post	△	F		Effect size Cohen's *d*
SBP (mmHg)	Exercise (n=12)	137.73 ±20.94	130.00 ±11.40	-7.73 ±17.97	Group	1.320	-0.37
					Time	0.026	
	Control (n=10)	138.10 ±19.03	144.70 ±14.96	6.60 ±13.13	Interaction	4.273	0.35
DBP (mmHg)	Exercise (n=12)	73.09 ±11.41	74.00 ±11.21	0.91 ±14.90	Group	0.012	0.08
					Time	0.401	
	Control (n=10)	72.70 ±6.72	75.10 ±7.16	2.40 ±7.43	Interaction	0.081	0.36
TC (mg/dL)	Exercise (n=12)	202.27 ±40.45	182.18$^{\#\#\#}$ ±30.22	-20.09 ±17.59	Group	1.401	-0.50
					Time	11.750**	
	Control (n=10)	208.60 ±26.81	207.00 ±22.34	-1.60 ±9.95	Interaction	8.538**	-0.06
TG (mg/dL)	Exercise (n=12)	113.82 ±45.80	109.18 ±36.96	-4.64 ±29.42	Group	1.126	-0.10
					Time	0.083	
	Control (n=10)	124.80 ±31.21	133.00 ±44.40	8.20 ±27.04	Interaction	1.077	0.26
HDL-C (mg/dL)	Exercise (n=12)	51.73 ±17.18	56.82$^{\#\#}$ ±19.30	5.09 ±6.50	Group	0.100	0.30
					Time	2.758	
	Control (n=10)	52.70 ±10.77	51.80 ±9.11	-0.90 ±4.84	Interaction	5.636*	-0.08

LDL-C (mg/dL)	Exercise (n=12)	127.15 ±42.26	97.80$^{###}$ ±20.84	-29.35 ±28.60	Group	2.126	-0.70
					Time	9.976**	
	Control (n=10)	130.94 ±27.00	128.60 ±21.74	-2.34 ±14.29	Interaction	7.246*	-0.09

Values are mean±standard deviation. SBP: systolic blood pressure, DBP: diastolic blood pressure, TC: total cholesterol, TG: triglyceride, HDL-C: high-density lipoprotein cholesterol, LDL-C: low-density lipoprotein cholesterol. $^{*}p < 0.05$, $^{**}p < 0.01$, $^{\#\#}p < 0.01$, $^{\#\#\#}p < 0.001$. Effect size range: |0.20|≦small<|0.50|<medium<|0.80|≦large.

Supplemental Table 4. Changes in insulin resistance after a 12-week aquatic exercise program

Variable	Group	Pre	Post	⊿	*F*		Effect size Cohen's *d*
Glucose (mg/dL)	Exercise (n=12)	111.55 ±23.47	94.00$^{\#\#}$ ±16.25	-17.55 ±23.33	Group	.800	-0.75
					Time	4.094	
	Control (n=10)	110.00 ±25.86	110.90 ±20.70	0.90 ±11.99	Interaction	5.027*	0.04
Insulin (μU/mL)	Exercise (n=12)	11.83 ±6.26	9.05 ±3.74	-2.78 ±6.56	Group	.100	-0.45
					Time	.014	
	Control (n=10)	8.27 ±6.26	11.32 ±4.66	3.05 ±3.11	Interaction	6.534*	0.49
HOMA-IR	Exercise (n=12)	3.36 ±2.02	2.11$^{\#}$ ±0.92	-1.26 ±1.96	Group	.001	-0.62
					Time	.687	
	Control (n=10)	2.39 ±2.06	3.08 ±1.32	0.69 ±0.96	Interaction	8.110*	0.34

Values are mean±standard deviation. HOMA-IR: homeostatic model assessment for insulin resistance. $^{*}p < 0.05$, $^{\#}p < 0.05$, $^{\#\#}p < 0.01$. Effect size range: |0.20|≦small<|0.50|<medium<|0.80|≦large.

Supplemental Table 5. Changes in aging-related sex hormones after a 12-week aquatic exercise program

Variable	Group	Pre	Post	△	*F*		Effect size Cohen's *d*
Estradiol (pg/mL)	Exercise (n=12)	5.46 ±0.72	5.72 ±1.19	0.25 ±1.41	Group	2.156	0.36
					Time	.166	
	Control (n=10)	5.71 ±0.68	5.26 ±0.42	-0.45 ±0.58	Interaction	2.156	-0.66
Testosterone (ng/mL)	Exercise (n=12)	0.08 ±0.04	0.06 ±0.04	-0.02 ±0.04	Group	.605	-0.50
					Time	16.052***	
	Control (n=10)	0.12 ±0.11	0.06## ±0.07	-0.06 ±0.05	Interaction	3.040	-0.55
DHEA-S (μg/dL)	Exercise (n=12)	62.76 ±24.46	67.65 ±23.46	4.89 ±8.74	Group	.168	0.20
					Time	.026	
	Control (n=10)	63.77 ±26.56	58.14 ±22.56	-5.63 ±12.12	Interaction	5.277*	-0.21
SHBG (nmol/L)	Exercise (n=12)	71.69 ±24.00	87.48## ±28.58	15.79 ±17.95	Group	.027	0.66
					Time	2.006	
	Control (n=10)	81.62 ±15.95	74.53 ±15.43	-7.09 ±7.73	Interaction	13.852**	-0.45

Values are mean±standard deviation. DHEA-S: dehydroepiandrosterone-sulfate, SHBG: sex hormone-binding globulin. *$p < 0.05$, **$p < 0.01$, ***$p < 0.001$, ##$p < 0.01$. Effect size range: |0.20|≦small<|0.50|<medium<|0.80|≦large.
